# A Fault Detection Method Based on an Oil Temperature Forecasting Model Using an Improved Deep Deterministic Policy Gradient Algorithm in the Helicopter Gearbox

**DOI:** 10.3390/e24101394

**Published:** 2022-09-30

**Authors:** Lei Wei, Zhe Cheng, Junsheng Cheng, Niaoqing Hu, Yi Yang

**Affiliations:** 1 College of Intelligence Science and Technology, National University of Defense Technology, Changsha 410073, China; 2 Laboratory of Science and Technology on Integrated Logistics Support, National University of Defense Technology, Changsha 410073, China; 3 College of Mechanical and Vehicle Engineering, Hunan University, Changsha 410073, China

**Keywords:** helicopter main gearbox, fault detection, oil temperature, deep deterministic policy gradient, data driven

## Abstract

The main gearbox is very important for the operation safety of helicopters, and the oil temperature reflects the health degree of the gearbox; therefore establishing an accurate oil temperature forecasting model is an important step for reliable fault detection. Firstly, in order to achieve accurate gearbox oil temperature forecasting, an improved deep deterministic policy gradient algorithm with a CNN–LSTM basic learner is proposed, which can excavate the complex relationship between oil temperature and working condition. Secondly, a reward incentive function is designed to accelerate the training time costs and to stabilize the model. Further, a variable variance exploration strategy is proposed to enable the agents of the model to fully explore the state space in the early training stage and to gradually converge in the training later stage. Thirdly, a multi-critics network structure is adopted to solve the problem of inaccurate *Q*-value estimation, which is the key to improving the prediction accuracy of the model. Finally, KDE is introduced to determine the fault threshold to judge whether the residual error is abnormal after EWMA processing. The experimental results show that the proposed model achieves higher prediction accuracy and shorter fault detection time costs.

## 1. Introduction

A helicopter is a kind of aircraft that can hover in the air, so it is widely used in the military, rescue, and transportation fields. Next, for the convenience of narration, Table 1 gives a detailed definition of the main acronyms. As the core component of the HTS, clearance, external force, friction and other factors interact with its dynamic behavior during the operation of the HMGB [1], making it deviate from the ideal operation states, leading to the occurrence of faults, and the failure of the gearbox is a direct threat to the flight safety of the helicopter. Therefore, the effective detection of gearbox failures that may cause catastrophic accidents is of great significance to ensure the flight safety of helicopters [2]. In order to satisfy the maintenance needs of helicopters, HUMS represented by EC135 [3], SA330 [4], AH-64, and UH-60 [5] is designed for health monitoring and fault diagnosis on key components such as HMGB and others, which has achieved obvious results in the reducing flight accident rate and maintenance costs. Generally, HUMS is composed of sensors for collecting vibration, sound, temperature, and other signals and a central computer for data processing and fault diagnosis [6].

Most researchers study the PHM of a HTS based on vibration signals. The vibration signals are processed and the fault types are recognized by using expert experience or machine learning methods [7,8,9]. Although the vibration signal of a helicopter carries rich running state information and can be used to detect early weak faults of mechanical components in time, HUMS cannot effectively and reliably diagnose some parts, such as the bearings of HMGB, from the vibration signal [10], because HMGB-status monitoring based on vibration signal analysis will encounter the following challenges: (1) The early fault features contained in the vibration signal is usually weak, and it will be interfered with by various noises in the transmission process. (2) The design of HMGB shows the trend of structural complexity. (3) HMGB is often quickly switched from different working states when performing multiple tasks, and the variable speed and load make it difficult to distinguish health and fault samples. (4) Given that a helicopter is high-reliability equipment, fault samples are extremely scarce; therefore, the distribution of relevant history data will be highly skewed to healthy samples.

In many cases, it is far more important to effectively and reliably detect HMGB anomalies than to identify specific fault types. Rashid et al. pointed out that any failure of HMGB would be reflected in the oil of the lubrication system, and the mechanical parts would not obtain ideal lubrication due to abnormal oil temperature, which would in turn accelerate the occurrence of micropitting, wear, scuffing, pitting, and other typical failures [11]. If the thermodynamic equation of HMGB can be known and can accurately calculate the oil temperature through the current working states, according to whether the actual oil temperature deviates from the normal predicted oil temperature, the ground crew can maintain the HMGB in time before a fault occurs. At present, the methods for establishing an oil temperature forecasting model can be mainly categorized into physics-based [12] and data-driven [13]. Using the physics-based method, Feng et al. derives relationships between oil temperature, transmission efficiency, rotational rate, and power output and believes that the oil temperature of the gearbox will increase linearly with power output and that, when the fault occurs, the transmission efficiency will inevitably decrease, resulting in the actual oil temperature being higher than the theoretical oil temperature [14]. However, with the increasing complexity of the equipment, using physics-based methods will involve a variety of parameters, making it difficult to accurately establish an oil temperature forecasting model.

In recent years, artificial intelligence technology has developed rapidly; therefore data-driven processes can rely on many algorithms to excavate the internal laws of historical data, such as the shallow perceptron, deep neural network, and statistical method, and has gradually become a means of verification to quantitatively describe the thermodynamic behavior of a gearbox. Data-driven processes use a large amount of health data to build the implicit mapping relationship between oil temperature and working state, and the residual errors between the actual value and the predicted value of oil temperature is used to evaluate the health degree of the gearbox through statistical methods. Liu et al. used the eXtreme gradient boosting (XGBoost) algorithm to establish the oil temperature regression forecasting model for condition monitoring WT gearbox [15]. Zeng et al. proposed sparse Bayesian learning to estimate the oil temperature of a WT gearbox [16]. Dhiman et al. utilized a twin support vector machine (TSVM) to predict the WT gearbox oil temperature, and an adaptive threshold is used to judge whether the gearbox is abnormal [17]. Wang et al. adopted a DNN-based framework to detect the health states of a wind turbine (WT) gearbox [18]. Guo et al. utilized an adam-trained LSTM to represent an oil temperature forecasting model to calculate the failure threshold [19]. Yang et al. combined the LSTM with a generalized regression neural network (GRNN) to form a weighted-combination oil temperature prediction model [20]. Jia et al. presented a robust denoising autoencoder (DAE) model to predict the raw temperature signal reconstruction error [21]. However, due to the increasingly complex structure of the HMGB and the increase in the number of sensors used for condition monitoring, the DL algorithm based on a single model or a hybrid model cannot meet the requirements. Therefore, intelligent state-of-the-art technology should be explored to improve the accuracy of HMGB oil temperature prediction under complex working states.

A model-free DRL framework integrates the perception of DL for the environment with the decision-making ability of RL, which can automatically find the optimal strategy through the reward of environmental feedback; hence, DRL has been successfully applied in thecomputer game [22], autonomous driving [23], machine vision [24], and fault diagnosis fields [25]. Although DRL is a promising technology, there are few references about research on DRL in gearbox-condition monitoring based on the oil temperature prediction principle. Up to now, only Liu et al. used the SARSA algorithm to select the features of each sub-series in gearbox condition monitoring, and it can be concluded that selecting appropriate features is conducive to improving the accuracy of oil temperature prediction [26]. However, the Q-learning algorithm, such as SARSA and DQB, cannot solve the time-series-prediction problem in high-dimensional and continuous state space [27]. In several commonly-used DRL frameworks, e.g., SARSA, DQN, advantage actor-critic (A2C), and DDPG, but DDPG is the only framework suitable for mapping the continuous state space to the corresponding continuous output action value, which can directly output the corresponding oil temperature value under the current working condition. In other to fill the research gap of the application of DRL in HMGB oil temperature forecasting and condition monitoring, an improved DDPG model for building a more accurate oil temperature forecasting model is proposed in this paper. The main contributions for the condition monitoring and health evaluation of HMGB in this paper, compared with the original DDPG algorithm, can be summarized as follows:

1. An improved DDPG framework is proposed, namely multi-CRDPG, in which CNN–LSTM is used as a basic learner to sense the input working condition information of HMGB; thereby, the strong feature extraction ability of CNN and the advantage of LSTM in dealing with time series prediction are combined. DDPG is introduced as an RL framework for training the basic learner, which enhances the prediction ability to deal with the complex oil temperature series of the basic learner.
2. A novel reward function is designed for educating the agent to output the predicted action as accurately as possible.
3. An explore strategy is presented, in which agent are encouraged to actively explore the unknown space in the early stage of training and use the learned experience to gradually converge on the ideal output action in the later stage of training.
4. In order to avoid the inaccurate estimation of the current state by a singer critic network and the inability to find the optimal strategy, a multi-critics network structure is advanced. A minimum and truncated mean processing method for a multi-critics network is conducive to reducing the deviation and variance of the estimated *Q*-value.
5. KDE is used to calculate the probability density function of the prediction residual errors in the healthy state of a HMGB to determine the failure threshold, and the trend of residual errors generated by EWMA control chart is to judge the HMGB health degree in the monitoring process.
6. The rest of this paper is organized as follows. Section 2 introduces the basic theories of the fault mechanism, RL, DDPG and CNN–LSTM algorithm. Section 3 provides the proposed multi-CRDPG algorithm. Section 4 describes the implementation details and experimental results ofthe mMulti-CRDPG in actual testing. Section 5 outlines conclusions and future works.

## 2. Basic Theories Involved

### 2.1. The Mechanism That Oil Temperature Can Reflect HMGB Health Degree

**Figure 1:**
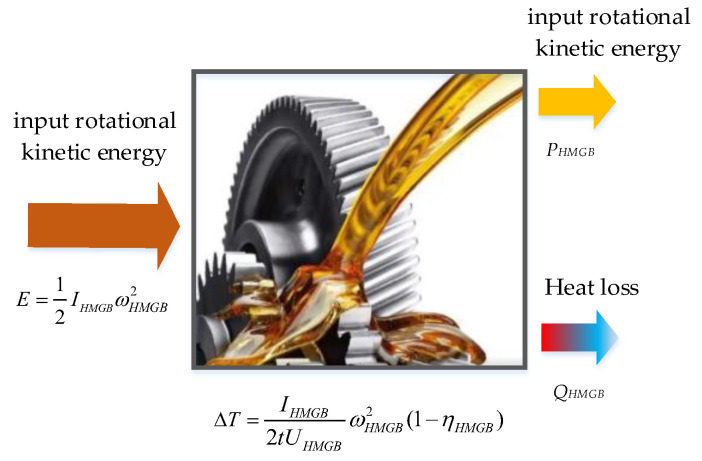
Energy flow direction of a HMGB.

HMGBs are subjected to the first law of energy conservation during operation, and the energy exchange is shown in Figure 1. As the carrier of heat exchange between a HMGB and the environment, the input energy loss of a HMGB can be related to the increase in oil temperature. The thermal energy balance equation of HMGB is expressed by Equation (1).

(1) $$E-Q_{HMGB}t=P_{HMGB}t$$

where *E* represents the input rotational kinetic energy of HMGB, *Q_HMGB_* represents the heat loss power of HMGB, *P_HMGB_* represents the output rotational kinetic power of the HMGB, and *t* represents running time.

The reason why HMGB generates extra heat during operation is the transmission efficiency, which is defined as:

(2) $$\eta_{HMGB}=\frac{P_{HMGB}t}{E}$$

Substituting Equation (2) into Equation (1) can be written as Equation (3):

(3) $$Q_{HMGB}t=(\frac{1}{\eta_{HMGB}}-1)P_{HMGB}t$$

Furthermore, *E* can be expressed in term of angular velocity and moment inertia:

(4) $$E=\frac{1}{2}I_{HMGB}\omega_{HMGB}^{2}$$

where *I_HMGB_* represents the moment inertia of HMGB, and $\omega_{HMGB}^{2}$ represents the angular velocity of HMGB.

Supposing the HMGB compound heat transfer coefficient is *U_GB_*, the oil temperature rise is Δ*T*, then the expression of Δ*T* is as follows:

(5) $$\Delta T=\frac{I_{HMGB}}{2tU_{HMGB}}\omega_{HMGB}^{2}{\left(1-\eta_{HMGB}\right)}_{}$$

From Equation (5), we can know that, when angular velocity is a fixed value, Δ*T* should decrease with the increase of transmission efficiency in unit time. When HMGB gradually deteriorates, its moment inertia and heat transfer coefficient are almost unchanged, and the transmission efficiency is significantly reduced [1]; the oil temperature rise at the state of HMGB failure is significantly higher than that at the natural state of a healthy HMGB.

### 2.2. The Concept of the Deep Deterministic Policy Gradient Algorithm

**Figure 2:**
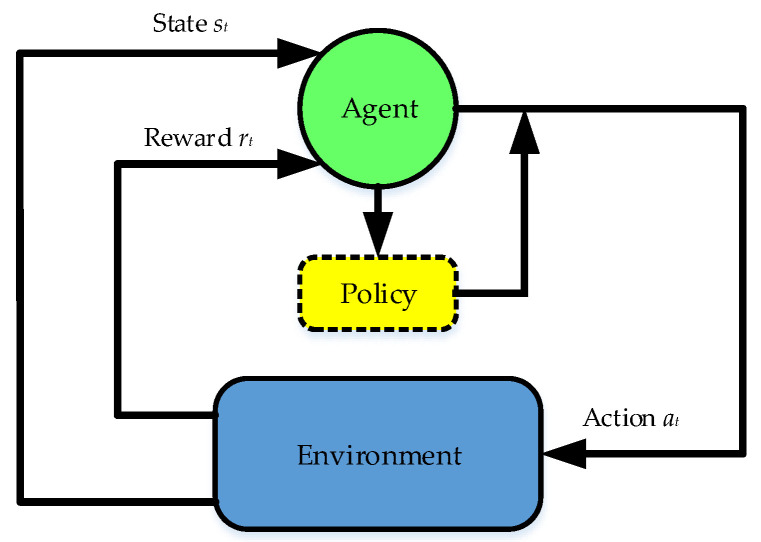
Reinforcement-learning closed-loop system.

RL is an important branch of machine learning. It can be represented as a closed-loop system composed of agents, environment, a state space *s_t_*∈*S*, an action space *a_t_*∈*A*, and a reward function *r_t_* = *R*(*s_t_*, *a_t_*, *s_t_*_+1_), as shown in Figure 2, in which the state space *s_t_*∈*S* describes the set of information received by the agent and the action space *a_t_*∈*A* describes the set of agents’ decision-making in state space *s_t_*∈*S* [28].

In RL, the interactive information between the agent and the environment can be described by a quadruple tuple (*s_t_*, *a_t_*, *r_t_*, *s_t_*_+1_), i.e., the next state *s_t_*_+1_ is just dependent on the current state *s_t_*, and the transition from the current state *s_t_* to the next state *s_t_*_+1_ can be regarded as the MDP. A complete time step of MDP is defined as τ = (*s*_0_, *a*_0_, *r*_0_),…, (*s_t_*, *a_t_*, *r_t_*), namely a trajectory. The return of a trajectory is the weighted sum of discount awards, which is calculated by Equation (6).

(6) $$R(\tau )=r_{0}+\gamma r_{1}+\gamma^{2}r_{2}+\cdots +\gamma^{T}r_{T}=\sum_{t=0}^{T}\gamma^{t}r_{t}$$

where *γ* is the discount factor, which reflects the impact of the current action *a_t_* on the future. *T* is the maximum time step in a trajectory.

Agents are trained to find an optimal strategy π^*^(*a_t_*|*s_t_*) to deal with state space *s_t_*∈S; the objective function strategy π(*a_t_*|*s_t_*) is the expected value of return on several trajectories, as shown in Equation (7).

(7) $$J(\tau )=E_{\tau \sim \pi }[R(\tau )]=E_{\tau }[\sum_{t=0}^{T}\gamma^{t}r_{t}]$$

Assuming that the agent performs actions according to a strategy π(*a_t_*|*s_t_*), the state value function *V_π_*(s) is used to evaluate the correlation degree of the current each state to the future state, and the state-action value function *Q_π_*(*s,a*) is used to evaluate the influence degree of each action in each state on the future state. The expressions of *V_π_*(*s*) and *Q_π_*(*s*,*a*) are shown in Equations (8) and (9), respectively.

(8) $$V_{\pi }(s)=E_{s_{0}=s,\tau \sim \pi }[\sum_{t=0}^{T}\gamma^{t}r_{t}]$$

(9) $$Q_{\pi }(s,a)=E_{s_{0}=s,a_{0}=a,\tau \sim \pi }[\sum_{t=0}^{T}\gamma^{t}r_{t}]$$

**Figure 3:**
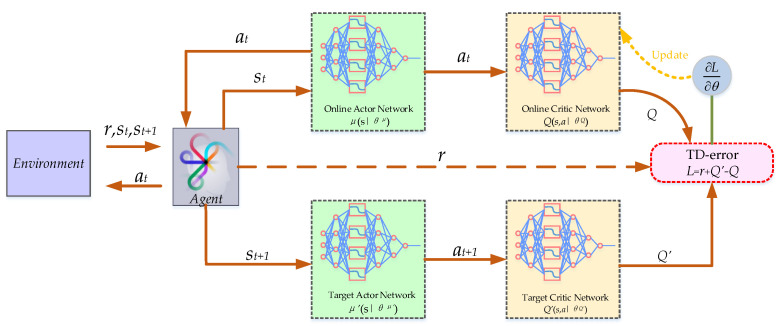
The basic framework of the DDPG.

In order to solve the optimal strategy π^*^(*a_t_*|*s_t_*) in continuous state space, i.e., maximize *V_π_*(s) and *Q_π_*(*s*,*a*), DDPG was presented by Google’s Deepmind team in 2016 [29]. The DDPG is one of the famous algorithms of DRL; it combines the deep neural network from DL with the Q-learning algorithm and the actor-critic structure from RL and includes four kinds of neural networks, namely a online actor network *μ*(s|*θ^μ^*), a target actor network *μ’*(s|*θ^μ’^*), an online critic network *Q*(*s,a*|*θ^Q^*) and a target critic network *Q’*(*s,a*|*θ^Q’^*), where *θ^μ^*, *θ^μ’^*, *θ^Q^* and *θ^Q’^* denotes the weight parameter of the four kinds of networks. The basic framework of the DDPG is shown in Figure 3.

The current state *s_t_* is the input to the actor network *μ*(s|*θ^μ^*) to obtain a deterministic output action *a_t_*, and the online critic network *Q*(*s,a*|*θ^Q^*) calculates the *Q*(*s,a*). However, one of the challenges of DDPG is that it is often difficult to make online actor network *μ*(s|*θ^μ^*) and critic network *Q*(*s,a*|*θ^Q^*) converge; therefore, it is necessary to introduce the target network *μ’*(s|*θ^μ’^*) and *Q’*(*s,a*|*θ^Q’^*) as a copy of the online network *μ*(s|*θ^μ^*) and *Q*(*s,a*|*θ^Q^*), respectively, which can temporarily fix the actor-critic network parameters to provide a reference for the update of the original network, so as to avoid the divergence of the original network after updating.

In addition, the DDPG is developed based on a deep Q-network (DQN), and the replay buffer is preserved, which is an important improvement to store historical transition tuples (*s_t_*, *a_t_*, *r_t_*, *s_t_*_+1_). The *Q*(*s,a*|*θ^Q^*) estimated by the target critic network is shown in Equation (10).

(10) $$y_{t}=r_{t}+\gamma Q^{\prime }(s_{t+1},\mu^{\prime }(s_{t+1}|\theta^{\mu^{\prime }})|\theta^{Q^{\prime }})$$

where *t* represents the sequence time of samples taken from the replay buffer.

**Figure 4:**
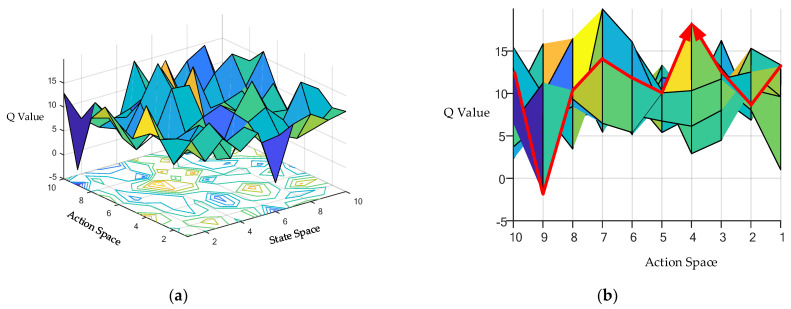
(**a**) A three-dimensional *Q*-table composed of *Q* values corresponding to each action in different states. (**b**) The *Q* value corresponding to each action in a certain state and finding the action corresponding to the maximum *Q* value through the gradient rising method.

Once the replay buffer is full of samples, sampling a batch of samples can be started to update the critic network and the actor network by minimizing Equation (11) and executing Equation (12), respectively. After that, the oldest samples will be squeezed out of the replay buffer by the new samples. Figure 4 shows that the actor network uses the gradient rise method to find the best output action *a_t_* corresponding to the state space *s_t_*∈*S,* i.e., the search for the optimal action with the largest *Q* value in a certain state.

(11) $$L_{critic,online}=\frac{1}{H}\sum_{i=0}^{H}{\left(y_{i}-Q(s_{i},a_{i}|\theta^{Q})\right)}^{2}$$

(12) $$\nabla L_{actor,online}=\frac{1}{H}\sum_{i=0}^{H}\nabla_{a}Q(s,a|\theta^{Q}){|}_{s=s_{i},a=\mu (s_{i})}\nabla_{\theta^{\mu }}\mu (s|\theta^{\mu }){|}_{s_{i}}$$

where *H* represents the maximum batch number of samples taken from the replay buffer.

Finally, soft assignment is used to steadily update the two target networks, as shown in Equations (13) and (14).

(13) $$\theta^{Q}=\tau \theta^{Q}+(1-\tau )\theta^{Q^{\prime }}$$

(14) $$\theta^{\mu^{\prime }}=\tau \theta^{\mu }+(1-\tau )\theta^{\mu^{\prime }}$$

where *α* represents the update speed, τ∈(0,1).

### 2.3. The Convolutional Long-Short Time Memory Neural Network

**Figure 5:**
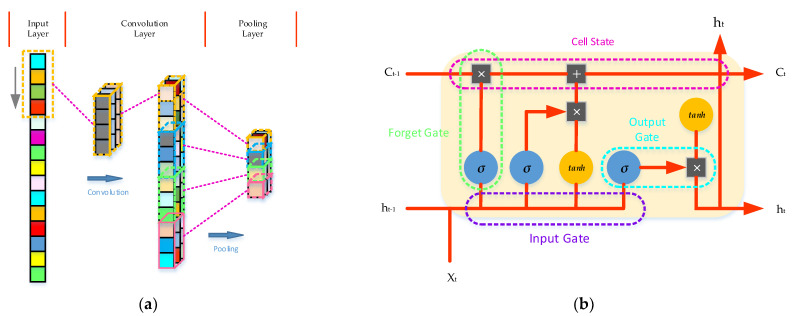
(**a**) The structure of a CNN network. (**b**) A cell of LSTM.

CNN–LSTM, as its name suggests, is a hybrid model of CNN and LSTM, which integrates the local feature extraction ability of CNN and the long-term and short-term prediction ability of LSTM [30]. Furthermore, 1-D CNN is applied to the feature extraction of oil temperature signal; Figure 5a shows its network structure, and 1-D convolution and pooling operation is its main calculation. When the data is sent to the convolution layer, the 1-D convolution kernel with customized length will slide on the data in order and perform convolution calculation. The output of the *i*-th 1-D convolution layer can be expressed as Equation (15). In the convolution layer, the data segment with the same length as the convolution kernel is dot-multiplied by a convolution vector, and then an offset term is added to output the operation result. All outputs calculated in each window will form a vector according to the convolution operation sequence, which is essentially filtering the signal. All signal segments share the same convolution kernel, which means that the signal is mapped and that the local features of the signal are extracted.

(15) $$\sigma ((f_{i}⊗h)(t)+b_{i})=\sigma (\sum_{k=-T}^{T}f_{i}(t)\cdot h(t-k)+b_{i}))$$

where *f_i_*(*t*) represents the convolution kernel whose size needs to be preset, and parameters are obtained by learning from input data. *h*(*t*) represents the input data. *b_i_* represents the bias factor. *σ*(·) represents the activation function; commonly used activation functions include Sigmoid, Relu, Tanh, etc. X_t_ h_t−1_ C_t−1_ h_t_ C_t_.

In the convolution process of signals, there is only a small amount of useful information, and most of the information is redundant. Adopting pooling processing can speed up the calculation speed and prevent overfitting, including max pooling, average pooling, etc.

For CNN–LSTM, the data will be input into the LSTM layer after the pooling operation. A LSTM network, as a variant of a recurrent neural network (RNN), can reflect the dependence of MDP by hiding the memory state, which is used to extract the medium- and long-term correlation characteristics of the corresponding time series from the stored CNN and reveal the essence of the time series. LSTM is composed of four neural network layers in a special connection mode. The gradient disappearance problem can be effectively solved through four interacting layers. Its structure (forget gate, input gate, update gate and output gate) is shown in Figure 5b [31]. The calculation process of a typical LSTM network unit module is shown in Equation (16).

(16) $$\{\begin{array}{c} i_{t}=\sigma (W_{i}h_{t-1}+U_{i}x_{t}+b_{i})\\ f_{t}=\sigma (W_{f}h_{t-1}+U_{f}x_{t}+b_{f})\\ o_{t}=\sigma (W_{o}h_{t-1}+U_{o}x_{t}+b_{o})\\ g_{t}=\tanh(W_{g}h_{t-1}+U_{g}x_{t}+b_{g})\\ C_{t}=C_{t-1}\cdot f_{t}+g_{t}\cdot i_{t}\\ h_{t}=\tanh(c_{t})\cdot o_{t} \end{array}$$

where *i_t_*, *f_t_*, *o_t_* and *g_t_* represent input gate, forget gate and output gate and, respectively, their activation functions *σ*(·) are sigmoid. *W_i_*, *W_f_*, *W_o_*, *U_f_*, *U_f_* and *U_o_* represent the weight matrix corresponding to the hidden state and the input state, respectively. *b_i_*, *b_f_*, *b_o_* and *b_g_* represent the bias term. *C_t_* represents the memory cell.

## 3. Improving DDPG for HMGB Condition Monitoring and Fault Detection

### 3.1. The Deign of Reward Incentive Function

Herein, the action of an agent is considered the predicted value of oil temperature. In the application of DDPG in oil temperature prediction, it is very important to design an appropriate reward function to guide the agent to accurately output the oil temperature value according to the input working conditions. According to previous research and experience, the response of an oil temperature signal to working states change has a certain delay; therefore, the oil temperature signal changes slowly. In a short time interval, the oil temperature value at the previous point will not differ much from that at the next point under a healthy-state HMGB. In the references about DDPG applied to forecast time series, the residual error between the output action *a_t_* and the actual load value is regarded as a reward function [32,33]. However, taking the residual error as the linear incentive condition is not only not sensitive to the agent at the initial stage of training, which slows down the training speed, but it is also too sensitive to the agent at the later stage of training, which results in difficulty in convergence. Based on this consideration, a new reward incentive function (RIF) is designed in Equation (17).

(17) $$\begin{array}{l} r_{t}=-k_{p}\exp(0.08\left|a_{t}-OT_{t}\right|)-k_{i}(\left|a_{t}-OT_{t}\right|-\sum_{t=0}^{T}\left|a_{t}-OT_{t}\right|/T)\\ -k_{d}[(a_{t}-OT_{t})-(a_{t-1}-OT_{t-1})] \end{array}$$

where *r_t_* is the RIF in time steps *t*. *T* means the number of samples that have been input into the model. *k_p_* is the proportion coefficient; *k_i_* is the integration coefficient, and *k_d_* is the differentiation coefficient. *OT_t_* and *OT_t_*_-1_ denote the actual oil temperature value at the time *t* and *t* − 1, respectively. *a_t_* and *a_t−1_* denote the predicted oil temperature value at the time *t* and *t* − 1, respectively.

**Figure 6:**
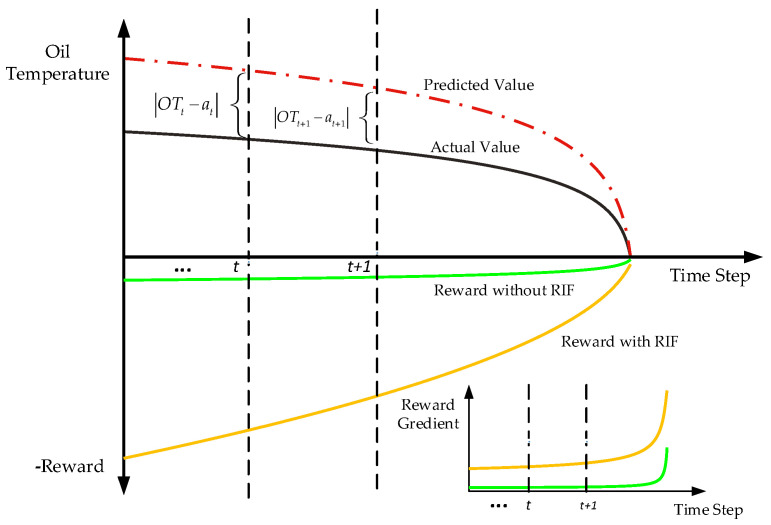
Schematic diagram of the RIF.

The first item can more sensitively detect the change of residual error and give greater punishment when the condition of the predicted value is larger than the actual value. The second item gives some positive rewards to the output actions *a_t_* that make the residual error less than the average residual error and some penalties to the output actions *a_t_* that make the residual error greater than the average residual error, making all the output actions *a_t_* more relevant, which is analogous to the inherent characteristic of the slow change of the oil temperature signal. The third item is considered to have an incentive effect, when the error (*a_t_* − *OT_t_*) at time *t* is smaller than the error (*a_t_*_-1_ − *OT_t_*_-1_) at time *t* − 1, and this trend should be rewarded [34]. Figure 6 shows how the designed RIF works. Gradient is used to measure the change of reward function. The gradient change of the RIF at time steps *t* and *t* + 1 is greater than that of the traditional reward function with the same error value change, which shows that the RIF is very sensitive to the change in error. RIF can stimulate agents towards output high-precision-prediction values better than traditional reward functions.

### 3.2. Variable Exploration Variance

Selecting the deterministic action *a_t_* corresponding to max *Q_π_*(*s_t_, a_t_*) in each state means that there will be many state-actions (*s, a*) that cannot be selected, which means that the agent cannot fully explore the entire continuous state space. Because *Q_π_*(*s_t_, a_t_*) is initialized randomly, it will lead to the inaccurate estimation of some *Q_π_*(*s_t_, a_t_*) without real experience. To avoid this challenge, an exploratory strategy is proposed, by adding a random number from a noise process *N*.

(18) $$\mu^{\prime }(s_{t})=\mu (s_{t}|\theta_{t}^{\mu })+N$$

where *N* can be any form of noise, and Gaussian noise is selected in this paper, denoted by *X~N*($\bar{x}$, *σ*^2^), as shown in Equation (19).

(19) $$F(x)=\frac{1}{\sqrt{2\pi }\sigma }\int_{-\infty }^{x}\exp(-\frac{{\left(x-\bar{x}\right)}^{2}}{2\sigma^{2}})dx$$

where $\bar{x}$ is the mean, and *σ* is the variance. Herein, $\bar{x}$ is set to 0.

**Figure 7:**
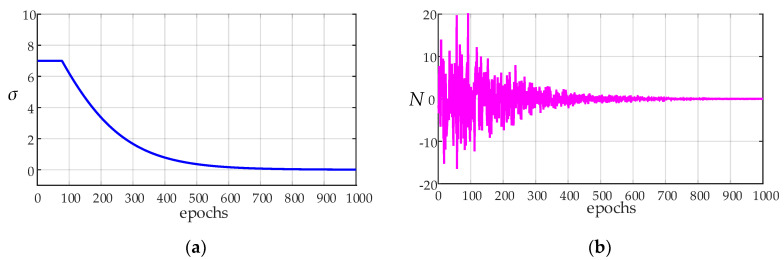
(**a**) The curve of variance *σ_epo_* decays as epochs increases. (**b**) The curve of noise *N*(*u*, $\sigma_{epo}^{2}$) decays as epochs increases.

Unfortunately, this kind of exploration is unstable. Simply adding noise to the output action may not be as effective as each output deterministic strategy. The key to finding the optimal strategy is to maintain the balance between exploration and output deterministic actions. A good solution is to add noise with high variance *σ* at the initial stage of training, so that the agent can quickly explore the state space. When the agent gradually learns a good strategy, it gradually reduces the variance *σ* of noise at a later stage of training so that the agent can output deterministic actions using previous experience. Therefore, an exploration strategy that the variance *σ* decreases with the increase of epoch is presented, as shown in Equation (20).

(20) $$\sigma_{epo}=\{\begin{array}{cc} 10-10\frac{\exp(\frac{4n_{epo}}{n_{epo\_total}})-\exp(-\frac{4n_{epo}}{n_{epo\_total}})}{\exp(\frac{4n_{epo}}{n_{epo\_total}})+\exp(-\frac{4n_{epo}}{n_{epo\_total}})} & \sigma_{epo}\leq 7\\ 7 & \sigma_{epo}>7 \end{array}$$

where *n_epo_* represents the current epoch, *σ_epo_* represents the current variance, and *n_epo_total_* represents the total epoch. For instance, when *n_epo_total_* = 1000, the variance *σ_epo_* and the noise *N*(*u*, $\sigma_{epo}^{2}$) decays as *n_epo_* increases, as shown in Figure 7.

### 3.3. Multi-Critics Networks Structure

In the training process, the DDPG algorithm updates the critic network based on the gradient rise method, and the performance of actor networks depends on critic networks. Similar to the maximization operation of DQN, there is an overestimation value problem in the evaluation of *Q_π_*(*s_t_, a_t_*) by the critic network. In the RL algorithm based on the value function, any small change in value estimation may lead to a suboptimal strategy. To solve this problem, Fujimoto et al. presents a twin delayed deep deterministic policy gradient (TD3) [35], and the algorithm adopts two critic networks and updates the critic network parameters by selecting a pair of the minimum state-action value *Q* as the target *Q* value, which alleviates the overestimation problem of DDPG to a certain degree. Supposing ${\hat{Q}}_{1}$ and ${\hat{Q}}_{2}$ represent the estimated action-state value from two independent critic networks, respectively, and that *Q*_real_ is the real value, there are two deviation terms *Z*_1_= ${\hat{Q}}_{1}$ − *Q*_real_ and *Z*_2_ = ${\hat{Q}}_{2}$ − *Q*_real_, which obey a uniform distribution, i.e., *Z_i_*_=1,2_*~U*(−*u*, *u*). In TD3, the expectation *E*[min *Z_i_*_=1,2_]=-*u*/3 and variance *Var*[min *Z_i_*_=1,2_] = 2*u^2^*/9 after the minimization operation, the negative expectation is introduced in each update of the critic network, resulting in the *Q* value being underestimated. This underestimation makes the *Q* value of critic networks lower than the real value, and the accumulated underestimation will generate suboptimal strategies, which will degrade the performance of the algorithm. In order to solve the problem of underestimation in TD3, an improved network structure based on the truncated mean of multi-critic networks is proposed.

The algorithm adds multiple critic networks to reduce the underestimation and estimation variance based on TD3, which can improve the performance and stability of the algorithm to a certain extent. Assuming that there are *K* deviations of critic networks (*K* > 5), and the deviation *Z_i_*_=1,2,…,*K*_ between their estimated action-state values ${\hat{Q}}_{i=1,\ldots ,K}$ and the real action-state value, *Q*_real_ follows the uniform distribution *U*(−*u*, *u*). Firstly, the highest action-state value *Qmax* and the lowest action-state value *Qmin* of multi-critic networks are removed to reduce the impact of extreme values on the real action-state value *Q*_real_. The truncated mean can better reflect the concentration trend of the deviation and reduces the upper and lower limits of the deviation, while the remaining *K* − 2 deviation *Z_i_*_=1,2,…,K-2_ follows a more concentrated uniform distribution *U*(−*u*_’_, *u*_’_) at this process, where *u*_’_< *u*. Second, taking any two critic networks to minimize, i.e., min *Q_i_*_=1,2_, and calculating the average value of min *Q_i_*_=1,2_ and *Q_i_*_=1,2,…,*K*-4_ from the remaining *K*-4 critic networks is a process described in Equation (21).

(21) $$\hat{Q}=\frac{1}{2}[\min Q_{i=1,2}+\frac{1}{K-4}(Q_{3}+Q_{4}+\cdots +Q_{K-4})]$$

where $\hat{Q}$ is the finally estimated action-state value.

The error value between the estimated action-state value $\hat{Q}$ and the actual action-state value *Q*_real_ is calculated in Equation (22). The estimation deviation of the multi-critic networks is lower than that of the TD3 algorithm. A lower estimation deviation helps to improve the stability of the algorithm and improve the performance of the algorithm.

(22) $$E(\hat{Q}-Q_{real})=E\left\{\frac{1}{2}[\min Z_{i=1,2}+\frac{1}{K-4}(Z_{3}+Z_{4}+\cdots +Z_{K-4})]\right\}=-\frac{1}{6}u^{\prime }$$

Furthermore, the variance of the error value $\hat{Q}-Q_{real}$ can be expressed as Equation (23). The results show that, compared with the TD3 algorithm, the estimated value obtained by the multi-critic networks is more stable. If the computer hardware allows it, increasing the number of critic networks is conducive to further reducing the variance of the estimated action-state value $\hat{Q}$.

(23) $$\begin{array}{ll} Var(\hat{Q}-Q_{real}) & =Var\left\{\frac{1}{2}[\min Z_{i=1,2}+\frac{1}{K-4}(Z_{3}+Z_{4}+\cdots +Z_{K-4})]\right\}\\ & =\frac{1}{4}E[(\min Z_{i=1,2}{)}^{2}]-\frac{1}{4}E[(\min Z_{i=1,2}){]}^{2}\\ & +\frac{1}{4{\left(K-4\right)}^{2}}Var(Z_{3}+Z_{4}+\cdots +Z_{K-4})\\ & =\frac{1}{18}{u^{\prime }}^{2}+\frac{1}{12(K-4)}{u^{\prime }}^{2} \end{array}$$

**Figure 8:**
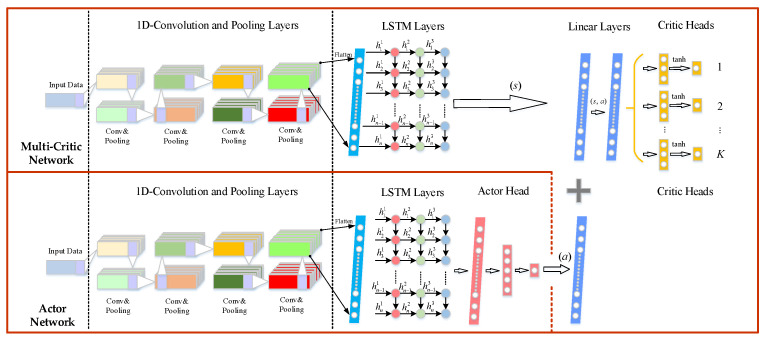
The structure of actor and critic networks.

Finally, in order to save computer memory, the multi-reviewer network structure will share some parameters. In addition, the actor network has a structure similar to that of the critic networks. After the actor network outputs the action *a_t_*, the action is subsequently transmitted to the full connection layer of the critic networks in the form of a sum, and finally the state-action value *Q_π_*(*s, a*) is obtained, as shown in Figure 8. To take into account the differences between the individual critic network and the overall performance of the multi-critic networks, a loss function with a weight and penalty mechanism is introduced [36], which is written in Equation (24). Table 2 shows the main process of the multi-CRDPG algorithm.

(24) $$\begin{array}{ll} L_{critic}= & a\frac{1}{H}\sum_{i=0}^{H}\sum_{k=1}^{K}{\left(r_{i}+\gamma Q_{k}(s_{i+1},a_{i+1}|\theta^{Q^{\prime }})-Q_{k}(s_{i},a_{i}|\theta^{Q})\right)}^{2}\\ & +b\frac{1}{H}\sum_{i=0}^{H}\sum_{k=1}^{K}{\left(r_{i}+\gamma \hat{Q}(s_{i+1},a_{i+1}|\theta^{Q^{\prime }})-Q_{k}(s_{i},a_{i}|\theta^{Q})\right)}^{2}\\ & +c\frac{1}{H}\sum_{i=0}^{H}\sum_{k=1}^{K}{\left(\hat{Q}(s_{i},a_{i}|\theta^{Q})-Q_{k}(s_{i},a_{i}|\theta^{Q})\right)}^{2} \end{array}$$

where *a, b* and *c* represent the weight coefficient, when *K* = 1, the loss function in this paper, degenerates into the loss function of original DDPG algorithm. 

### 3.4. The Condition Monitoring and Fault Detection for HMGBs Based on Multi-CRDPG and EWMA

**Figure 9:**
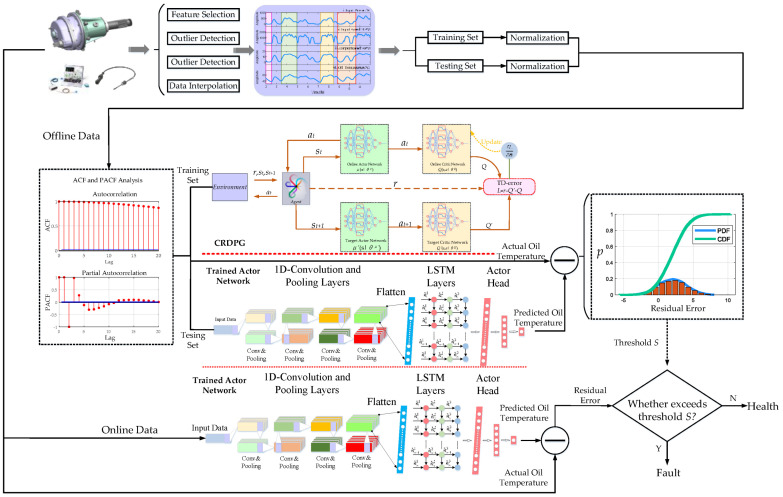
The diagram of condition monitoring and fault detection for a HMGB.

A condition monitoring and fault detection method for HMGB based on multi-CRDPG and EWMA by using SCADA data is proposed in this section, and an overall diagram is shown in Figure 9.

It can be observed that the diagram mainly consists of four steps: feature selection, data processing, offline data training and online fault detection. Their detailed explanations are as follows.

Feature selection: The first task in constructing an oil temperature forecasting model is to select high-quality input features. In SCADA data, although all the features have a little correlation with the increase of oil temperature, the differences include strong correlations and weak correlations. The agent may be forced to learn the relationship between these weak correlation features and oil temperature, so as to establish an unstable model, which is not conducive to forecasting the new data. The input characteristics carrying useful information and selecting features with strong correlation can effectively prevent the over-fitting of the basic learner. Therefore, it is necessary to eliminate the weak correlation features. In this paper, a cross-correlation function (CCF) is used to measure the correlation between input characteristics and oil temperature at different times to solve lag time series analysis, as written in Equation (25).

(25) $$CCF_{i}=\int_{-\infty }^{+\infty }f_{i}(t)f_{OT}(t+\tau )$$

where *f*_OT_(*t* + *τ*) represents the oil temperature series, and *f_i_*(*t*) represents the different input features series.

Data processing: In most of the data-driven method for oil temperature forecasting, the actual data may contain missing and abnormal values, and the scales of each parameter are also different. Hence, outlier detection, missing value interpolation and data normalization is an indispensable operation in data processing. Firstly, for the slowly changing data such as oil temperature, abnormal points refer to those sudden change points. In practice, it is impossible for the difference between the data value at time *t* and the data value at time *t* + 1 to be large, which is a simple and effective outlier detection method. In order to restore the authenticity and objectivity of information, if *OT_t_* is considered an abnormal point, the mean between *OT_t−1_* and *OT_t+1_* is used to substitute for *OT_t_*. Secondly, to deal with a small number of missing values, in addition to optimizing the acquisition system as much as possible to avoid missing data, bezier interpolation can also be used to obtain high-precision and consecutive data in this study. Finally, normalizing the data of different scales is beneficial in training the agent, which can adjust the eigenvalues of the input data to a similar range and facilitate the selection of a uniform learning rate.

Offline data training: The multi-CRDPG algorithm is used to establish an oil temperature forecasting model in this step. In the feature-extraction stage, an autocorrelation function (ACF) and a partial autocorrelation function (PACF) are adopted to determine the optimal lag period. Before training the agent, the SCADA data should be divided into a training set and a testing set by a series of time windows, each windows containing several input series with a length equal to the lag period and a prediction series with a time step of *n*. After initializing the parameters, the training task can be executed.

Fault threshold calculation: Because of the accuracy limitation of the prediction model, the residual error between the predicted oil temperature and the oil temperature in the test set can be used to determine the fault threshold. The residual error processed by EWMA can not only reflected the trend of the residual value but can also effectively eliminate the false alarm point, so that the fault threshold can be set more scientifically, EWMA expression is shown in Equation (26).

(26) $$e_{t}=(1-\lambda )e_{t-1}+\lambda {\bar{e}}_{t}$$

where *e_t_* and *e_t−1_* represents the residual error at time *t*, and ${\bar{e}}_{t}$ represents the sliding average of the residual error at time *t*. λ represent the weight of historical data; it reflects the impact of the previous data on the next data; this impact will be gradually weakened with the passage of time.

A thorny problem here is that the distribution of the generated residual error is unknown, and the residual errors generated by different models are considered to have different distributions, e.g., *T*-distribution, Gaussian distribution, etc. Unlike parameter estimation, nonparametric estimation does not add any prior knowledge but fits the distribution according to the characteristics and properties of the data itself, which can obtain a better model than parameter estimation. KDE is a nonparametric estimation method. Without knowing the distribution of residual error, the fault threshold can be expressed as Equation (27).

(27) $$S=\frac{1}{N}\sum_{t=0}^{N}e_{t}+S_{th}$$

where *S* represents the fault threshold, and *N* represents the number of data points in testing set. *S_th_* represents an interval upper limit. According to the interval estimation theory in statistics, the distribution characteristics of residual errors can be analyzed by KED. Assuming that the residual error is distributed in the interval [0, *S_th_*] with a probability value of 1−*p*, 1−*p* is called the confidence level, which represents the cumulative probability distribution. The smaller the value of *p*, the less likely the occurrence of *S* > *S_th_*. By setting different probability values for *p*, multiple thresholds of *S* can be obtained to judge the health degree of a HMGB.

Online fault detection: After the oil temperature forecasting model is established and the fault threshold *S* is set, the real-time working condition data can be input into the model. By comparing whether the difference in the residual error between the predicted oil temperature value and the actual oil temperature value exceeds the fault threshold *S*, whether the HMGB is degraded can be detected.

## 4. Experimental Verification and Results Analysis

**Figure 10:**
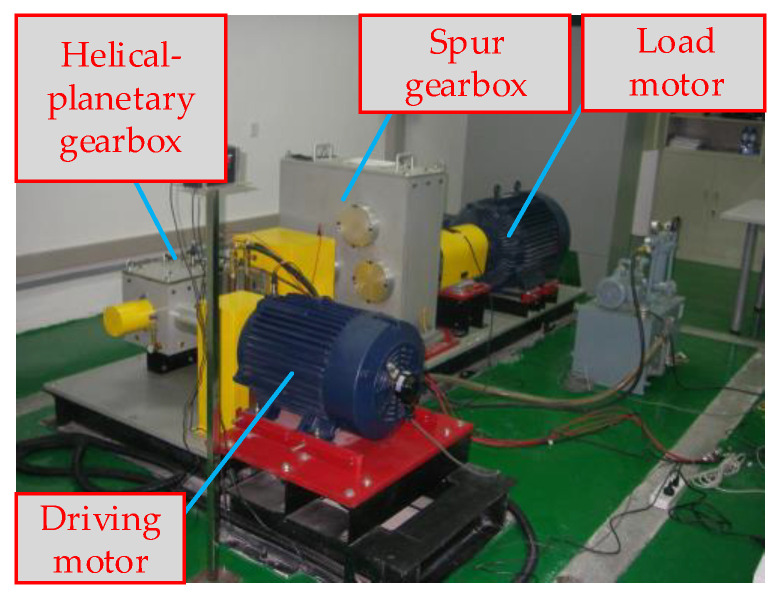
Simulated helicopter transmission system for the condition monitoring and diagnosis detection of a HMGB.

**Figure 11:**
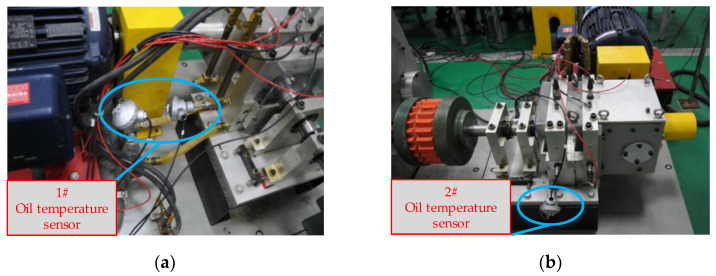
Arrangement position of 1# and 2# temperature oil sensors. (**a**) Gearbox inlet oil temperature sensor. (**b**) Gearbox oil temperature sensor.

In this section, to verify the effectiveness of the proposed fault detection method for a HMGB, a simulated helicopter transmission system is manufactured to collect the data generated under a healthy state to train the oil temperature forecasting model and carry out a series of fault-seeded experiments. The test rig mainly includes a drive motor, HPGB, spur gearbox, load motor, and data acquisition system, as shown in Figure 10. In this study, HPGB is the monitored object, and the collected variables include load torque, driving motor speed, gearbox oil pressure, gearbox inlet oil temperature, gearbox oil temperature and ambient temperature, and gearbox oil temperature; the gearbox oil temperature reflects the health status of HPGB. The 1# sensor for collecting the inlet oil temperature and the 2# sensor for collecting the gearbox oil temperature are shown in Figure 11.

### 4.1. The Establishment of an Oil Temperature Forecasting Model

#### 4.1.1. Generation of Datasets

**Figure 12:**
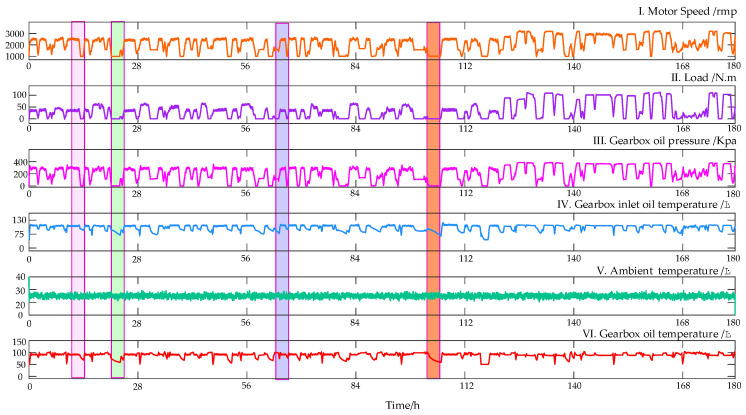
Visualization of the variables collected in HPGB.

In actual flight, the helicopter cannot change the engine power by adjusting the throttle. The output power is approximately constant and controls the lift by changing the angle of the rotor hub, but the HMGB speed is variable. Therefore, the condition of constant motor output power is simulated, and the motor output power is 53 KW. The relevant variables were collected by a sampling interval of 1 s, from 17 June 2022, to 25 June 2022, totaling 180 h and 648,000 points. Figure 12 shows the results after data preprocessing; then it can be observed that the gearbox oil temperature has a certainly delayed correlation with other variables but has little relationship with the ambient temperature. The dataset details and the maximum cross-correlation coefficient between each variable and the gearbox oil temperature are shown in Table 3. The motor speed, load, gearbox oil pressure and gearbox inlet oil temperature has the strongest CCF value with the gearbox oil temperature. Therefore, the above four variables are selected as inputs for the oil temperature forecasting model.

**Figure 13:**
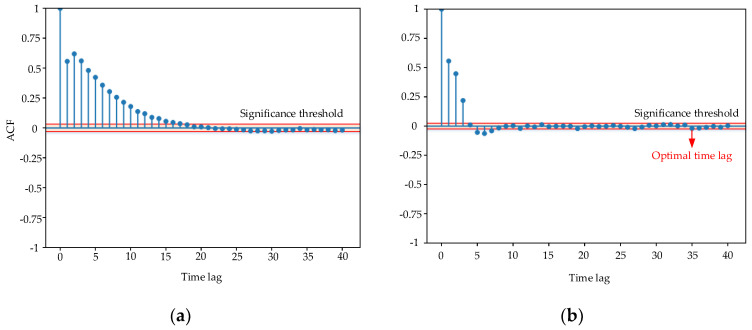
ACF and PACF of oil temperature. (**a**) ACF diagram. (**b**) PACF diagram.

In this study, after constructing the collected data into a dataset, the first 70% is set as the training set, and the remaining 30% is set as the test set. The lag time is selected by analyzing ACF and PACF. Figure 13 shows the ACF and PACF diagrams of the oil temperature. The red line represents the significance threshold, which is limited to 5% within the scope of this case. The ACF diagram tails off to zero, while the PACF diagram represents a truncation trend. In PACF, the time steps before 35 exceed this threshold, which is regarded as heavily relevant to the oil temperature, so the optimal time lag period is set to 35. It is worth noting that, although single-step was is selected in this paper, in order to ensure the accuracy of oil temperature prediction, it was still decided to use the first 35 time steps to predict the oil temperature at the next time.

**Figure 14:**
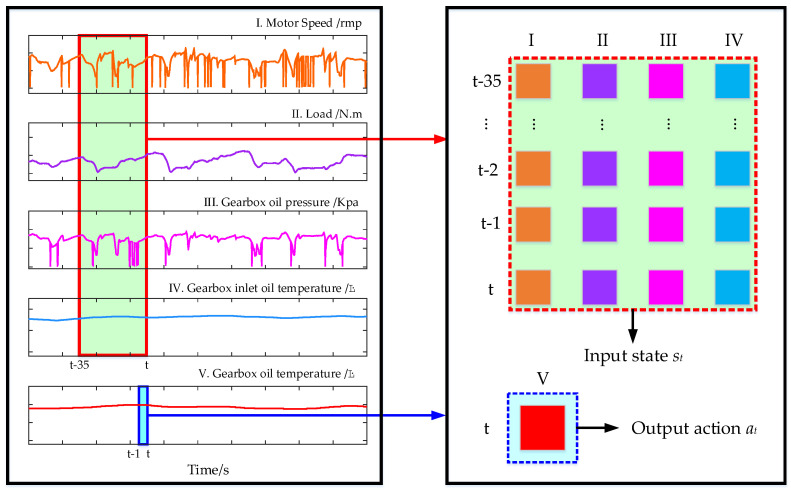
Schematic diagram of feature samples.

It is an important step to apply the proposed multi-CRDPG to the establishment of oil temperature prediction model in this paper, and the prediction of oil temperature should be changed to the continuous control of DRL. Before training an agent, the specific state *s_t_* and action *a_t_* at each time should be clearly defined. Feature samples are generated by using two data windows, in which state *s_t_* is composed of four time series with 35 timesteps, and action *a_t_* is only composed of output oil temperature *OT_t_* at time *t*, as shown in Figure 14. After winnowing, the training set contained 12,960 samples, and the testing set contained 5554 samples. Once the oil temperature prediction model is converted into the decision-making process of the current HMPG working condition, the multi-CRDPG algorithm can be used.

Parameter optimization is an indispensable step to ensure the performance of the oil temperature prediction model, and the random grid search method is applied as the parameter tuning method in this paper due to the model involving too many hyperparameters. The random grid search method abandons the global hyperparameter space, instead selecting some parameter combinations to constructs the hyperparameter subspace. Compared with the enumeration grid search method, the random grid search method requires less computation. For example, assuming that there are parameters A and B in the 2-D search space, then the value of A is [1,2,3,4,5,6,7]; the value of B is the [1,2,3,4,5,6,7], and the search step is set to 1. Then the enumeration grid search method must search all 49 parameter combinations, but the random grid search method only needs to select some parameter space values as parameter combinations to search. Although the results of the random grid search method are uncertain, the minimum loss is very close to the minimum loss obtained by the enumeration grid search method; the random grid search method is used together with cross validation, mainly using K-fold cross-validation. The main idea is to divide the original datasets into K groups; take a verification set for each sub-datasets and use the remaining K-1 sub-datasets as the training set, so that K trained models can be obtained; and take the average error of K times as the final evaluation index. The K is set to 10 in this paper. In addition, within the limitation of each sample length (35 data points), increasing the convolution layers and pooling layers of convolution neural network model as much as possible enhances the feature extraction ability. Detailed experimental conditions and parameter settings are shown in Table 4.

#### 4.1.2. Performance Evaluation of the Model

Only by using reasonable indices to evaluate the performance of the model can it be correctly evaluated and persuasive; therefore, the four classical evaluation indices were used in this paper, i.e., mean absolute error (MAE), *R*-square (*R*^2^), mean absolute percentage error (MAPE) and root mean squared error (RMSE). Different indicators can reflect the performance of the model from different perspectives, and four indicators can comprehensively evaluate the model. The expressions are written in Equations (28) to (31).

(28) $$MAE=\frac{1}{N}\sum_{t=0}^{N}\left|OT_{t}^{actual}-OT_{t}^{predicted}\right|$$

(29) $$R^{2}=1-\left[\sum_{t=0}^{N}{\left(OT_{t}^{actual}-OT_{t}^{predicted}\right)}^{2}/\sum_{t=0}^{N}{\left(OT_{t}^{actual}-\sum_{t=0}^{N}OT_{t}^{predicted}/N\right)}^{2}\right]$$

(30) $$MAPE=\frac{1}{N}\sum_{t=0}^{N}\left|(OT_{t}^{actual}-OT_{t}^{predicted})/OT_{t}^{actual}\right|$$

(31) $$RMSE=\sqrt{\frac{1}{N}\sum_{t=0}^{N}{\left(OT_{t}^{actual}-OT_{t}^{predicted}\right)}^{2}}$$

where $OT_{t}^{actual}$ and $OT_{t}^{predicted}$ indicate the actual and predicted values at time step *t,* respectively. It is worth noting that smaller the value of MAE, MAPE and RMSE and bigger the value of *R*^2^, the higher the prediction accuracy of the model.

#### 4.1.3. Performance Comparison of Different Models

This subsection compares multi-CRDPG, CRDPG (only using CNN–LSTM as a basic learner of the DDPG algorithm, without RIF and multi-critic), RDPG, DDPG and other baseline models in terms of prediction accuracy, generalization and robustness. As we all know, the convergence time of the DRL algorithm is always longer than that of the DL algorithm, so the multi-CRDPG is only compared with RDPG and DDPG in terms of time cost. The existing models include classical models and state-of-the-art models. The other baseline models include the classical models LS-SVM, NARX, ARIMA and the state-of-the-art models CNN, GRU and CNN–LSTM, and they have been proven effective in the prediction of time series. In order to gradually analyze the advantages of the method proposed in this paper, the parameters of GRU, CNN, CNN–LSTM, DDPG and CRDPG are the same as those of the corresponding modules in multi-CRDPG. The parameter settings of the remaining models, LS-SVM, NARX and ARIMA, are described as below, and detailed parameters are shown in Table 5.

(1) LS-SVM: Penalty coefficient (C), kernel coefficient (gamma);
(2) NARX: Input felays (ID), feedback delays (FD), hidden size (N);
(3) ARIMA: AR/auto-regressive(p), MA/moving average (q), integrated(d)

**Figure 15:**
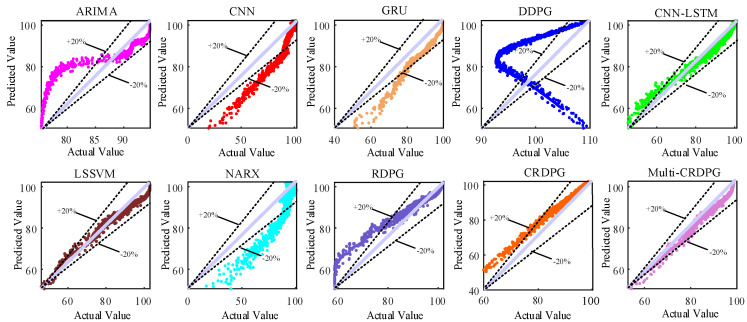
Oil temperature prediction results of ten forecasting models.

**Figure 16:**
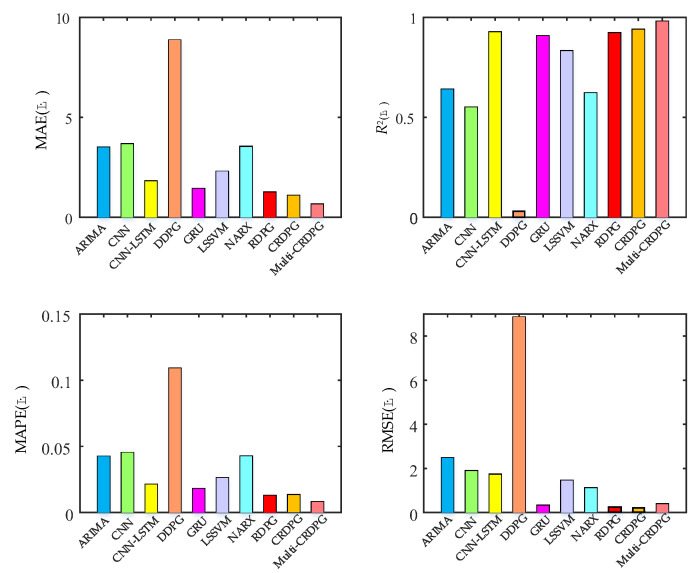
MAE, *R*^2^, MAPE and RMSE results of ten forecasting models.

Next, 10 oil temperature prediction experiments were carried out and the average of relevant results was taken. Figure 15 and Figure 16 display the oil temperature prediction results of the ten models under different working conditions in testing sets, and the comparison results can be summarized in detail as follows:

(a) Some conventional time series prediction models, including LSSVM, NARX, ARIMA and GRU, can predict the change trend in oil temperature to a certain extent, but in the face of complex working condition data, the ability of the above models to improve the prediction accuracy is very limited. As a classical feature extraction algorithm, CNN is good at data classification, but it is not good at excavating the transformation rules of time series, so its prediction performance is unsatisfactory. Of three DRL models, DDPG performs the worst among these models, even worse than conventional DL, because the structure of the BP neural network is too simple.
(b) The key to the successful application of the DRL algorithm is to choose a model with excellent performance as the basic learner. After extracting the feature of the time series, the prediction accuracy is higher than directly forecasting from the original data, and the training time of the model is shorter due to the simplification of the sequence information. By observing four evaluation indicies of LSSVM, NARX, ARIMA, CNN, GRU and CNN–LSTM, CNN–LSTM performed better than other models, which implies it has more potential as a basic learner of DRL and obtains optimal predicted accuracy.
(c) When comparing GRU and CNN–LSTM with their corresponding RL algorithms RDPG and CRDPG, it was fully proven that the performance of the basic learner guided by the reinforcement learning framework has been greatly improved. The possible reason is that the decision-making ability of the reinforcement learning framework is stronger than that of the traditional deep learning method for directly fitting data.

**Figure 17:**
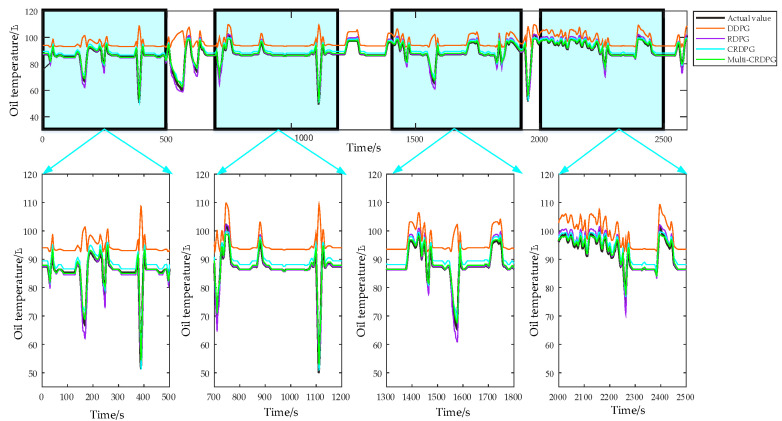
The prediction performance of DDPG, RDPG, CRDPG and multi-CRDPG in the testing set.

In order to further verify the effectiveness of the proposed improved method in terms of time cost and prediction accuracy, it is necessary to compare multi-CRDPG with DDPG, CRPG and CRDPG. Figure 17 shows the forecasting results (only showing the results of the last 2592 s) of the above four DRL algorithms. It can be observed that CRDPG obviously outperform the RDPG and DDPG models, which indicates that using CNN–LSTM as a basic learner can effectively perceive time series information compared with GRU and BP. RDPG cannot correctly predict the trend in oil temperature in the time period when the working conditions change sharply, and the prediction error is unacceptable for the actual fault detection of HMGBs. However, the predicted result of CRDPG is always higher than the actual oil temperature, and the evaluating indicators of CRDPG measured by MAE, RMSE, R^2^ and RMSE are 1.09 °C, 0.94 °C, 1.27 °C and 0.04 °C, respectively, which is caused by the overestimation of the state value of the working condition by a single critic network. When multi-critic is introduced in multi-CRDPG, the MAE, RMSE, R^2^ and RMSE are 0.66 °C, 0.98 °C, 0.008 °C and 0.4 °C. In addition to RMSE, other indicators have been greatly improved, which proves that the multi-critic network can correctly estimate the state value of the working condition.

**Figure 18:**
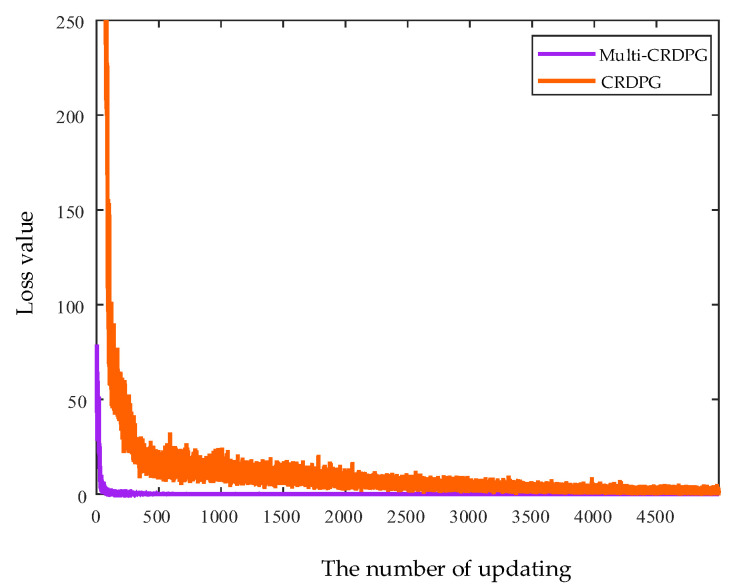
The loss value of CRDPG and multi-CRDPG in the training process.

Figure 18 presents the loss value of the CRDPG and multi-CRDPG in the training process, and it can be intuitive to see that the loss value of the multi-CRDPG decreases rapidly near the 100th updating time and have converged before the 500th updating time, but the loss value of CRDPG decreases slowly. This indicates that the designed RIF has a strong incentive effect on the training basic learner.

**Figure 19:**
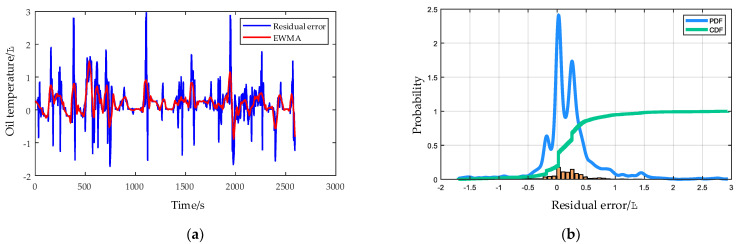
(**a**) The residual error of the HPGB oil temperature in the healthy state and after EWMA processing. (**b**) The distribution of residual error.

The residual error of the HPGB oil temperature in a healthy state is shown in Figure 19a. Although some residual error values exceed 3 °C, the overall distribution is symmetrical, with an average value of 0.198 °C and a standard deviation of 0.483 °C. The residual error processed by the EWMA control principle is shown by the red line in Figure 19a. Figure 19b shows the distribution of residual error. Setting 99.5% confidence, according to probability density function (PDF) and cumulative distribution function (CDF), results in a residual error of 2.61 °C being determined as the fault threshold.

### 4.2. Fault Detection Analysis

In order to explore the actual performance of the proposed fault detection method based on an multi-CRDPG oil temperature forecasting model and an EWMA control chart, a series of damage-seeded experiments was performed with different fault types in the transmission system and lubricating oil system of the HPGB. The occurrence of these faults will cause the oil temperature to rise. Naturally, the variable composition of the data set is the same as that of the data set for establishing the oil temperature prediction model in the actual test. The three damage-seeded experiments are tested in this paper, including planet gear broken teeth (Fault 1), a damaged bearing cage and rolling elements (Fault 2) and a clogged oil filter element (Fault 3). The detailed experimental environment is shown in the Table 6.

#### 4.2.1. Planet Gear Broken Teeth

**Figure 20:**
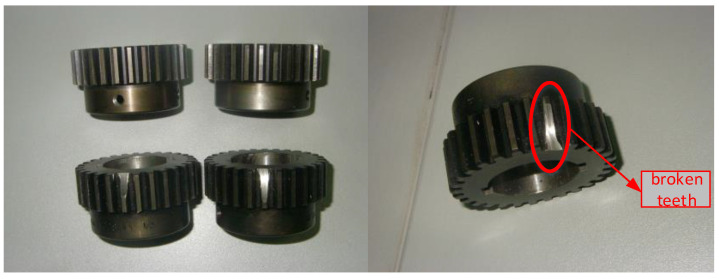
Broken-teeth-seeded planet gear.

**Figure 21:**
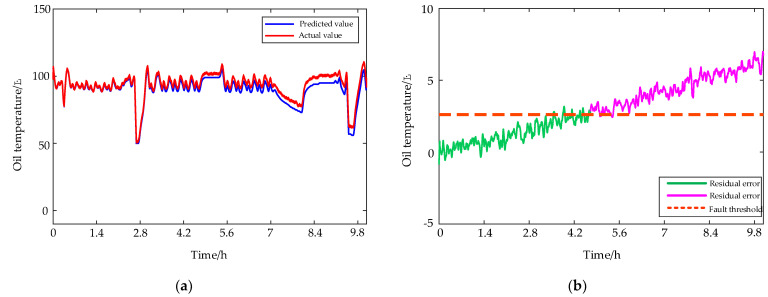
(**a**) Predicted and actual values of oil temperature under planet gear broken teeth condition. (**b**) The EWMA control chart of residual errors between the predicted and actual values of oil temperature.

As shown in Figure 20, planetary gear tooth breakage is a typical fault that threatens the operation safety of a HMGB, and it must be detected as soon as possible. When this fault is seeded, the predicted and actual oil temperature values are shown in Figure 21a, and the EWMA control chart of residual errors between predicted and actual oil temperature values is shown in Figure 21b. The green and pink lines represent residual errors below and above the fault threshold, respectively. Since the broken tooth has little influence on the operation state of HPGB in the early stage, it can hardly cause the oil temperature to rise, so the fault cannot be detected. As time goes on, the transmission system of the HPGB gradually deteriorates due to the planet gear broken teeth, and the oil temperature tends to rise. After the fault was seeded for about 18,000 s, if the actual oil temperature was higher than the predicted oil temperature and higher than the fault threshold 2.61 °C, then the HPGB is deemed to have a serious fault.

#### 4.2.2. Damaged Bearing Cage and Rolling Elements

**Figure 22:**
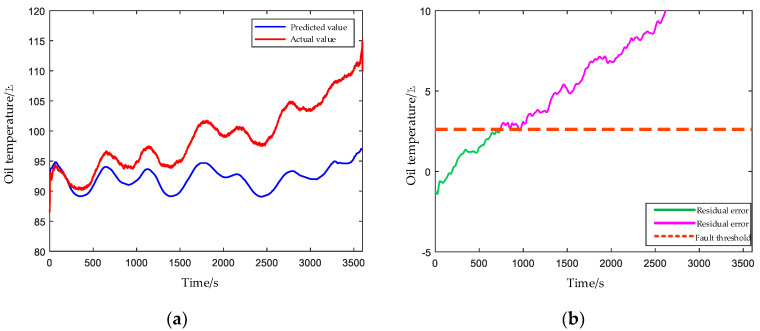
(**a**) Predicted and actual values of the oil temperature under the bearing with a broken cage and rolling element. (**b**) The EWMA control chart of residual errors between the predicted and actual values of oil temperature.

Bearing is also an important part in the transmission system, and its cage and rolling element are broken, which are common failures and will lead to other failures. Figure 22 shows the predicted and actual oil temperature value after the bearing with broken cage and rolling element are seeded in HPGB. In this experiment, the driving motor was kept in the high-speed range, which accelerated the degradation of the bearing, resulting in a rapid rate of oil temperature rise. The oil temperature exceeded the fault threshold 900 s after the fault was seeded.

#### 4.2.3. Clogged Oil Filter Element

**Figure 23:**
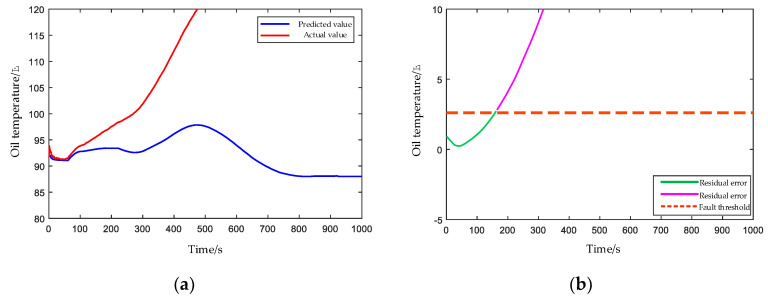
(**a**) Predicted and actual values of the oil temperature under the clogged oil filter element condition. (**b**) The EWMA control chart of residual errors between predicted and actual values of oil temperature.

In addition to the transmission system, the lubrication oil system is also an important system in the gearbox, which can ensure the lubrication and heat dissipation of mechanical parts. In order to simulate the clogging of the oil filter element, impurities are added into the lubricating oil artificially, and the filter element is rendered clogged. It can be observed from Figure 23 that the actual oil temperature is lower than the predicted oil temperature due to the influence of the ambient temperature at the beginning of the HPGB operation. When impurities accumulate on the oil filter element to a certain extent, the filter element will gradually become blocked, and the poor oil supply of the gearbox will lead to difficulty in heat dissipation and oil temperature rise. When it exceeds the threshold line at about 162 s, the HPGB is judged to be abnormal.

Finally, a comparison of the time costs for the above different models detects faults under experiments repeated 30 times is showed in Table 7. The proposed multi-CRDPG model requires the shortest time in three fault detection cases, and it can be concluded that the better the performance of the oil temperature forecasting model, the earlier the seeded fault can be found. Table 8 shows the missing rate for the above different models to detect faults within the duration time under all experiments. Apparently, multi-CRDPG and CRDPG have high reliability, and all seeded faults were identified in repeated experiments. Other models have a certain number of missing alarms for different faults, and, with the reduction of the fault severity, the probability of a missing alarm increases.

## 5. Conclusions and Future Works

Accurate oil temperature forecasting has great significance for the fault detection of HMGBs due to it being necessary to establish a forecasting model to describe the relationship between the HMGB working condition and oil temperature in the healthy state; this model will compare whether the residual error between the predicted oil temperature corresponding to the current working condition and the actual oil temperature exceeds the fault threshold. Conversely, an inaccurately predicted oil temperature can result in false alarms and missed alarms.

In this paper, a novel fault detection method based on an improved deep deterministic policy gradient algorithm with a CNN–LSTM-based learner, reward incentive function and multi-critic networks, and an EWMA control chart is proposed for oil temperature forecasting and fault detection. Actual HPGB datasets includes health samples and three failure cases; nine baseline and four evaluation indicators are used to verify the performance of the proposed model. According to the results of many comparison experiments, the five conclusions are summarized as follows:

(1) The proposed model has the advantages of higher prediction accuracy and more stable convergence than other baseline models. The results of comparison experiments in the datasets of each working condition demonstrate that an accurate oil temperature prediction model is successfully established. Meanwhile, the robustness of the model is verified, which can ensure the reliability of the prediction and detection results.
(2) The proposed deep deterministic policy gradient method is based on a CNN–LSTM network, which can extract complex time series features, eliminate redundant information, reduce noise influence and excavate the change rules of time series. Moreover, CNN–LSTM educated by a deep deterministic policy gradient framework can obtain better performance than the original CNN–LSTM.
(3) The proposed reward incentive function can accelerate and stabilize the convergence of model training by exciting the agent, which is worth being rewarded at different time steps.
(4) The proposed variable exploration variance is beneficial for the agent to fully explore the state space and correctly evaluate each state value in the initial training stage. Reduce the noise variance at the later training stage to make the model converge gradually.
(5) The proposed multi-critic network structure and a state-action value estimation strategy can reduce the overestimation and underestimation of the state-action values of the agent to improve the forecasting accuracy of the basic learner, which is a key step to further improve the prediction accuracy of the model.

Although the proposed model has the above innovations and advantages, it has some limitations that require further improvement. Firstly, the model involves many hyperparameters, and it needs to select a good initial value to ensure the prediction accuracy of the model. Therefore, an adaptive parameter selection method needs to be designed in the future. Finally, there is a risk of overfitting the proposed model with the increase of iteration times. In the future, an updated method should be developed to ensure that the model is updated in the direction of better performance.

## Figures and Tables

**Table 1:** Detailed definitions of the main acronyms.

Acronym	Detailed Definition
HMGB	Helicopter main gearbox
HTS	Helicopter transmission system
HUMS	Health and usage monitoring system
DRL	Deep reinforcement learning
RL	Reinforcement learning
DL	Deep learning
CNN–LSTM	Convolutional long-short time memory
LSTM	Long short-term memory
CNN	Convolutional neural network
DDPG	Deep deterministic policy gradient
RDPG	Recurrent deterministic policy gradient
GRU	Gate recurrent unit
KDE	Kernel density estimation
EWMA	Exponentially weighted moving average
HPGB	Helical gear pair-two-stage planetary gearbox
MDP	Markov decision process

**Table 2:** The main process of the multi-CRDPG algorithm.

Multi-CRDPG
1: Initialize the parameters *θ^μ^*, *θ^μ’^* of online actor network *μ*(s|*θ^μ^*) and online critic networks *Q*(*s,a*|*θ^Q^*), and their target network *μ’*(s|*θ^μ’^*), *Q’*(*s,a*|*θ^Q’^*) is set to *θ^μ ’^*= *θ^μ^, θ^Q’^* = *θ^Q^*. 2: Initialize the experience replay buffer *B,* the batch size *H* and the number of critic output *K* 3: Set the learning parameters *α*, *β*, *τ*, *n_epo_total_* and weight coefficient *a, b, c*. 4: for episode *n_epo_* = 1, …, *n_epo_total_* do: 5: for *t* = 1,2,…, *training size* do: 6: Receive initial state *s*_1_ 7: Calculate the current noise variance *σ_epo_* according to equation (20) 8: Select action *a_t_* = *μ*(s|*θ^μ^*) + *N*(0, *σ_epo_*) from the actor network *μ*(s|*θ^μ^*) 9: Execute action *a_t_*, get reward *r_t_* and receive the next state *s_t_*_+1_ 10: Store transition (*s_t_*, *a_t_*, *r_t_*, *s_t_*_+1_) in *B* 11: Sample random batch size of *H* transitions from *B* 12: Calculate the loss function *L_critic_* and the estimated *Q* value: Calculate the estimated state-action $\hat{Q}(s_{t},a_{t}|\theta^{Q^{\prime }})$ of the target critic network using Equation (21) Minimize the loss *L_critic_* using Equation (24) Update the parameters of the online critic network: $\theta_{k}^{Q}=\theta_{k}^{Q}-\alpha \nabla L_{critic}$ 13: Calculate the gradient of the $\hat{Q}(s_{t},a_{t}|\theta^{Q^{\prime }})$ for the online actor network by using Equation (12) Update the parameters of the online actor network: $\theta^{\mu }=\theta^{\mu }-\beta \nabla L_{actor}$ 14: Update the parameters of the target actor network and target critic network using soft updating *θ^μ’^* = (1 − *τ*)*θ^μ^* , *θ^Q’^* = (1 − *τ*)*θ^Q’^* 15: end for 15: end for

**Table 3:** Relevant acquisition variables.

Acquisition Variables	Unit	Mean	Std	Max Normalized CCF
Motor speed	rpm	1114.1	620.0	0.8897
Load	N.m	36.6	34.0	0.7381
Gearbox oil pressure	Kpa	214. 8	114.5	0.8965
Gearbox inlet oil temperature	°C	69.2	20.1	0.9915
Ambient temperature	°C	25.0	0.98	0.4123
Gearbox oil temperature	°C	88.7	20.8	1

**Table 4:** The detailed experimental conditions and parameter settings of the multi-CRDPG algorithm.

Modules	Layers	Types	Parameters	Input/Output Channel
Critic Network	1	Convolution	KS:11 S:1 P:5	4/16
	2	Pooling layer	PS:4	16/16
	3	Convolution	KS:3 S:1 P:1	16/32
	4	Pooling layer	PS:4	32/32
	5	LSTM	N:512 HN: 5	32/4
	6	Linear layer	N: 512	512/128
	7	Linear layer	N: 128	128/64
	8	Linear layer	N: 64	64/1
	…	…	…	…
	27	Linear layer	N: 512	512/128
	28	Linear layer	N: 128	128/64
	29	Linear layer	N: 64	64/1
Actor Network	1	Convolution	KS:11 S:1 P:5	4/16
	2	Pooling layer	PS:4	16/16
	3	Convolution	KS:3 S:1 P:1	16/32
	4	Pooling layer	PS:4	32/32
	5	LSTM	N:512 HN: 5	32/4
	6	Linear layer	N: 512	512/128
	7	Linear layer	N: 128	128/64
	8	Linear layer	N: 64	64/1
Parameters: α = 0.00005, β = 0.00005, τ = 0.05, *B* = 1000, *H* = 32, *K* = 8, *n_epo_total_* = 10, *a* = 0.5*, b* = 0.5*, c* = 0.1, λ = 0.9, k*_p_* = 2.2, k*_i_* = 1.8, k*_d_* = 0.5.Hardware and software: CPU: Intel Core i5-11400F K, GPU: GeForce RTX 1650s, Programing language: python, Deep learning framework: pytorch.				

KS: Kernel size; PS: Pooling size; S: stride; P: padding; N: nodes; AN: Actor network; HN: Hidden layers number.

**Table 5:** The detailed parameters of LS-SVM, NARX and ARIMA.

Models	Detailed Parameters
LS-SVM	C = 8, gamma = 0.02
NARX	ID = 35, FD = 1, N = 512
ARIMA	P = 16, q = 10, d = 1

**Table 6:** The detailed experimental environment.

Projection	Duration Time	Number of Tests	Ambient Temperature
Fault 1	36,000s	30	23~28 °C
Fault 2	3600s	50	23~28 °C
Fault 3	1000s	70	23~28 °C

**Table 7:** Time costs for different models to detect faults.

Models	Fault 1	Fault 2	Fault 3
Multi-CRDPG	18,002 ± 40.3 s	900 ± 20.7 s	159 ± 10.4 s
CRDPG	18,502 ± 34.2 s	933 ± 19.5 s	172 ± 13.9 s
RDPG	18,937 ± 40.5 s	1012 ± 21.2 s	200 ± 9.9 s
DDPG	24,557 ± 64.2 s	2321 ± 50.3 s	323 ± 33.2 s
LS-SVM	19,423 ± 44.8 s	1134 ± 24.3 s	235 ± 11.3 s
NARX	20,032 ± 23.2 s	1342 ± 23.2 s	283 ± 23.2 s
ARIMA	19,623 ± 37.1 s	1216 ± 23.4 s	252 ± 16.6 s
CNN	20,144 ± 53.8 s	1385 ± 42.2 s	299 ± 27.4 s
GRU	19,002 ± 37.1 s	1022 ± 26.7 s	211± 14.6 s
CNN–LSTM	18,722 ± 37.2 s	961 ± 23.5 s	184 ± 20.2 s

**Table 8:** Missing rate for different models to detect faults.

Models	Fault 1	Fault 2	Fault 3
Multi-CRDPG	0.0%	0.0%	0.0%
CRDPG	0.0%	0.0%	0.0%
RDPG	3.3%	2.0%	0.0%
DDPG	26.7%	16.7%	5.7%
LS-SVM	6.6%	6.0%	2.9%
NARX	6.6%	6.0%	2.9%
ARIMA	6.6%	6.0%	2.9%
CNN	26.7%	8.0%	4.3%
GRU	13.3%	2.0%	1.4%
CNN–LSTM	6.6%	2.0%	0.0%

## References

[1] Hu J., Hu N., Yang Y., Zhang L., Shen G. (2022). Nonlinear dynamic modeling and analysis of a helicopter planetary gear set for tooth crack diagnosis. Measurement.

[2] Sun C., Wang Y., Sun G. (2020). A multi-criteria fusion feature selection algorithm for fault diagnosis of helicopter planetary gear train. Chin. J. Aeronaut..

[3] Bolvashenkov I., Kammermann J., Herzog H.G. Electrification of Helicopter: Actual Feasibility and Prospects. Proceedings of the 2017 IEEE Vehicle Power and Propulsion Conference (VPPC).

[4] Kamble S.B., Desai V., Jeppu Y.V., Prajna System Identification for Helicopter Longitudinal Dynamics Model—Best Practices. Proceedings of the 2015 International Conference on Industrial Instrumentation and Control (ICIC).

[5] Bayoumi A., Ranson W., Eisner L., Grant L.E. Cost and effectiveness analysis of the AH-64 and UH-60 on-board vibrations monitoring system. Proceedings of the 2005 IEEE Aerospace Conference.

[6] Song J.H., Yang J.W., Rim M.S., Kim Y.Y., Kim J.H., Park H., Seok J.N., Kim C.G., Lee H.C., Choi S.W. Implementation of Sensor-embedded Main Wing Model of Ultra Light Airplane for Health and Usage Monitoring System (HUMS) Test-bed. Proceedings of the 2012 12th International Conference on Control, Automation and Systems (ICCAS).

[7] Wu J.Y., Sun C., Zhang C., Chen X.F., Yan R.Q. (2022). Deep clustering variational network for helicopter regime recognition in HUMS. Aerosp. Sci. Technol..

[8] Li T.F., Zhao Z.B., Sun C., Yan R.Q., Chen X.F. (2020). Adaptive Channel Weighted CNN With Multisensor Fusion for Condition Monitoring of Helicopter Transmission System. IEEE Sens. J..

[9] Marple S.L., Marino C., Strange S. Large dynamic range time-frequency signal analysis with application to helicopter Doppler radar data. Proceedings of the Sixth International Symposium on Signal Processing and its Applications.

[10] He D., Bechhoefer E. Development and validation of bearing diagnostic and prognostic tools using HUMS condition indicators. Proceedings of the 2008 IEEE Aerospace Conference.

[11] Rashid H.S.J., Place C.S., Mba D., Keong R.L.C., Healey A., Kleine-Beek W., Romano M. (2015). Reliability model for helicopter main gearbox lubrication system using influence diagrams. Reliab. Eng. Syst. Safe.

[12] Modaresahmadi S., Khalesi J., Li K., Bird J.Z., Williams W.B. (2020). Convective heat transfer analysis of a laminated flux focusing magnetic gearbox. Therm. Sci. Eng. Prog..

[13] Rashid H., Khalaji E., Rasheed J., Batunlu C. Fault Prediction of Wind Turbine Gearbox Based on SCADA Data and Machine Learning. Proceedings of the 2020 10th International Conference on Advanced Computer Information Technologies (ACIT).

[14] Feng Y.H., Qiu Y.N., Crabtree C.J., Long H., Tavner P.J. (2013). Monitoring wind turbine gearboxes. Wind Energy.

[15] Liu Y.R., Wu Z.D., Wang X.L. (2020). Research on Fault Diagnosis of Wind Turbine Based on SCADA Data. IEEE Access.

[16] Zeng X.J., Yang M., Bo Y.F. (2020). Gearbox oil temperature anomaly detection for wind turbine based on sparse Bayesian probability estimation. Int. J. Electr. Power Energy Syst..

[17] Dhiman H., Deb D., Muyeen S.M., Kamwa I. (2021). Wind Turbine Gearbox Anomaly Detection Based on Adaptive Threshold and Twin Support Vector Machines. IEEE Trans. Energy Convers..

[18] Wang L., Zhang Z.J., Long H., Xu J., Liu R.H. (2017). Wind Turbine Gearbox Failure Identification with Deep Neural Networks. IEEE Trans. Ind. Inform..

[19] Guo R.J., Zhang G.B., Zhang Q., Zhou L. Early Fault Detection of Wind Turbine Gearbox Based on Adam-Trained LSTM. Proceedings of the 2021 The 6th International Conference on Power and Renewable Energy.

[20] Yang S.Y., Zheng X.X. Prediction of Gearbox Oil Temperature of Wind Turbine Based on GRNN-LSTM Combined Model. Proceedings of the 2021 The 6th International Conference on Power and Renewable Energy.

[21] Jia X.J., Han Y., Li Y.J., Sang Y.C., Zhang G.L. (2021). Condition monitoring and performance forecasting of wind turbines based on denoising autoencoder and novel convolutional neural networks. Energy Rep..

[22] Silver D., Huang A., Maddison C.J., Guez A., Sifre L., van den Driessche G., Schrittwieser J., Antonoglou I., Panneershelvam V., Lanctot M. (2016). Mastering the game of Go with deep neural networks and tree search. Nature.

[23] You C.X., Lu J.B., Filev D., Tsiotras P. (2019). Advanced planning for autonomous vehicles using reinforcement learning and deep inverse reinforcement learning. Robot. Auton. Syst..

[24] Dogru O., Velswamy K., Huang B. (2021). Actor-Critic Reinforcement Learning and Application in Developing Computer-Vision-Based Interface Tracking. Engineering.

[25] Ding Y., Ma L., Ma J., Suo M.L., Tao L.F., Cheng Y.J., Lu C. (2019). Intelligent fault diagnosis for rotating machinery using deep Q-network based health state classification: A deep reinforcement learning approach. Adv. Eng. Inform..

[26] Liu H., Yu C.Q., Yu C.M. (2021). A new hybrid model based on secondary decomposition, reinforcement learning and SRU network for wind turbine gearbox oil temperature forecasting. Measurement.

[27] Guo M.Z., Liu Y., Malec J. (2004). A new Q-learning algorithm based on the Metropolis criterion. IEEE Trans. Syst. Man Cybern. Part B.

[28] Luo F., Zhou Q., Fuentes J., Ding W.C., Gu C.H. (2022). A Soar-Based Space Exploration Algorithm for Mobile Robots. Entropy.

[29] Ma Y.P., Zhu W., Benton M.G., Romagnoli J.A. (2019). Continuous Control of a Polymerization System with Deep Reinforcement Learning. J. Process Control.

[30] Wang Z.J., Cui J., Cai W.A., Li Y.F. (2022). Partial Transfer Learning of Multidiscriminator Deep Weighted Adversarial Network in Cross-Mechine Fault Diagnosis. IEEE Trans. Instrum. Measurement.

[31] He X.X., Wang Z.J., Li Y.F., Svetlana K., Du W.H., Wang J.Y., Wang W.Z. (2022). Joint decision-making of parallel machine scheduling restricted in job-machine release time and preventive maintenance with remaining useful life constrains. Reliab. Eng. Syst. Saf..

[32] Liu T., Xu C.L., Guo Y.B., Chen H.X. (2019). A novel deep reinforcement learning based methodology for short-term HVAC system energy consumption prediction. Int. J. Refrig..

[33] Liu T., Tan Z.H., Xu C.L., Chen H.X., Li Z.F. (2020). Study on deep reinforcement learning techniques for building energy consumption forecasting. Energy Build..

[34] Zhang W.Y., Chen Q., Yan J.Y., Zhang S., Xu J.Y. (2021). A novel asynchronous deep reinforcement learning model with adaptive early forecasting method and reward incentive mechanism for short-term load forecasting. Energy.

[35] Fujimoto S., van Hoof H., Meger D. (2018). Addressing Function Approximation Error in Actor-Critic Methods. Int. Conf. Mach. Learn..

[36] Fan J.J., Chen J.P., Fu Q.M., Lu Y., Wu H.J. (2021). DDPG algorithm based on multiple exponential moving average evaluation. Comput. Eng. Design.

